# Construction of pain prediction model for patients undergoing hepatic arterial chemoembolization

**DOI:** 10.1186/s12957-023-02986-y

**Published:** 2023-03-20

**Authors:** Ping-Wei Song, Ye-Hui Liu, Tao Wang, Lei Yu, Jing-Li Liu

**Affiliations:** 1 Surgical Department 1, Yantai Yuhuangding Hospital, Yantai, 264000 China; 2 Department of Interventional Therapy, Yantai Yuhuangding Hospital, Yantai, 264000 China; 3 Catheterization Room, Yantai Yuhuangding Hospital, No. 20 Yuhuangding East Road, Zhifu District, Yantai, 264000 China

**Keywords:** Carcinoma; Hepatocellular, Transcatheter arterial chemoembolization, Pain, Risk factors, Prediction model

## Abstract

**Objective:**

To construct a predictive model for pain in patients undergoing hepatic arterial chemoembolization (TACE) in interventional operating room.

**Methods:**

Through literature review and expert interviews, a questionnaire was prepared for the assessment of pain factors in patients with hepatic arterial chemoembolization. A prospective cohort study was used to select 228 patients with hepatic arterial chemoembolization in a tertiary and first-class hospital. The data of the patients in the pain group and the non-pain group were compared, and a rapid screening prediction model was constructed by univariate analysis and logistic regression analysis, and its prediction effect was tested.

**Results:**

Tumor size, liver cancer stage, and chemoembolization with drug-loaded microspheres and pirarubicin hydrochloride (THP) mixed with lipiodol were independent predictors of pain in patients after hepatic arterial chemoembolization. Finally, the pain prediction model after TACE was obtained. The results of Hosmer–Lemeshow test showed that the model fit was good (*χ*^2^ = 13.540, *p* = 0.095). The area under the receiver operating characteristic curve was 0.798, *p* < 0.001.

**Conclusion:**

The rapid screening and prediction model of pain in patients undergoing hepatic arterial chemoembolization has certain efficacy, which is helpful for clinical screening of patients with high risk of pain, and can provide reference for predictive pain management decision-making.

## Introduction

Transcatheter arterial chemoembolization (TACE) is the first-line treatment for primary liver cancer, but post-embolization pain is a common accompanying symptom [1]. Pain is an unpleasant emotional experience [2]. Studies have shown that the incidence of moderate to severe pain in patients with primary liver cancer after TACE is 59.3 to 85.5% [3–5]. The study by Andersen et al. [6] showed that pain was the primary cause of prolonged hospital stay after TACE. There are many factors that affect pain after TACE in patients with liver cancer, but there are no literature reports on the evaluation tools, prediction models, standard preventive measures, and management systems of pain after TACE. Moreover, some patients’ pain control is not ideal in clinical pain management, which may be related to the lack of effective communication between medical staff and patients [7]. If we can comprehensively evaluate the influencing factors of pain in patients early, effectively predict the degree of postoperative pain, and adopt the strategies of preoperative pre-intervention and timely intraoperative pain relief, the postoperative response of patients can be effectively reduced. Therefore, an information technology-based assessment tool for rapid screening of TACE patients at high risk of pain needs to be developed. This study intends to construct a rapid screening and prediction model for pain in patients with TACE, in order to provide a reliable assessment tool for clinical prevention of pain in patients with TACE.

## Materials and methods

### Research objects

Consulting experts: This study selected 16 experts from 5 cities in Shandong Province. Selection criteria: (1) the research field is interventional therapy, (2) master degree or above, (3) deputy senior title or above, (4) at least 10 years of experience in the field of interventional therapy, and (5) participate in this research voluntarily.

Evaluation objects: all consecutive patients with liver cancer TACE who were admitted to the interventional therapy department of a tertiary hospital in Yantai from May 2020 to September 2020 were screened. The diagnostic criteria for liver cancer were based on the “Guidelines for the diagnosis and treatment of primary liver cancer (2019 edition)” [8]. The inclusion criteria were patients who (1) were aged ≥ 18 years, (2) were pathologically or clinically diagnosed as hepatocellular carcinoma, (3) had an Eastern Cooperative Oncology Group (ECOG) score of 0–2, (4) were Child–Pugh liver function grade A or B, or (5) were China Liver Cancer Staging (CNLC) Ib–IIIb. Exclusion criteria: (1) obvious abdominal pain symptoms before surgery, (2) a history of long-term use of analgesics, and (3) prophylactic use of analgesics before and during TACE. The details of TACE procedure were as follows: (1) conventional TACE: the tumor blood supply artery was embolized with iodized oil and pirarubicin emulsion to slow down the blood flow in the tumor blood supply artery, and then the gelatin sponge particles or embolization pellet with the same diameter as the blood supply artery was used to supplement the embolization to slow down the blood flow. (2) DEB-TACE: evenly mixed DEB and pirarubicin were slowly injected into the feeding artery of HCC by pulse injection for embolization. The protocol of this study was approved by our institutional review board (approval number no. 20200107) and all patients signed their informed consents at their enrolment.

### Research methods

The questionnaire was designed to retrieve relevant literature at home and abroad in the past 5 years. The key words were liver cancer, hepatic arterial chemoembolization, pain, and influencing factors. A total of 57 target literatures were screened, and 10 causes of pain in TACE patients with the highest frequency were found in the literature, including age, gender, history of chronic liver disease, tumor location close to the liver capsule, tumor size, number of tumors, the dose of doxorubicin greater than 50% of the total theoretical dose, the amount of lipiodol embolism, the use of CalliSpheres® drug-loaded microspheres for TACE, and whether pirarubicin hydrochloride (THP) mixed with lipiodol is used. It is organized into three modules: basic information of patients, basic characteristics of tumors, and TACE treatment methods. Then, 16 experts are invited to conduct 2 rounds of consultation by referring to the Delphi method. When the experts are concentrated in the academic conference organized by the province, they are invited. The paper questionnaire was filled out, and experts who were not present used the form of questionnaire stars to summarize and organize expert opinions to determine the final draft of the questionnaire for the evaluation of pain factors in TACE patients. A total of 3 primary indicators were constructed, including basic information of patients, basic characteristics of tumors, and TACE treatment methods; 15 secondary indicators were included. The content validity index of 16 experts was 0.978.

In the pre-trial stage of clinical application, 15 TACE patients who met the inclusion and exclusion criteria were selected, and the measured Cronbach’s coefficient was 0.887. In the clinical application evaluation stage, 228 TACE patients who were hospitalized in the interventional therapy department of the hospital from June 2020 to October 2020 were selected, and 228 cases were effectively evaluated.

We determined the origin of the pain mainly based on nature type, symptoms, and timing of occurrence. When measuring the pain during surgery and within 24 h after surgery, a ruler about 10 cm in length and the visual analog scale (VAS) [9] was used, with 10 scales on one side and “0” and “10” on both ends. “0” indicated no pain, and “10” represented the most severe pain. In clinical use, the side with the scale was turned away from the patient, and the patient was asked to mark the corresponding position on the ruler that can represent his or her pain level. The nurse assigned a score to the patient by looking at the number behind the position marked. According to the VAS pain scale, 0–3 was considered as mild pain, which did not affect sleep, and 4 was considered as moderate pain, which had slightly affected sleep. Therefore, a VAS of greater than 4 was considered as the presence of pain and patients with VAS ≥ 4 were included in the post-TACE pain group.

### Statistical methods

SPSS 25.0 statistical software was used for data analysis. The enumeration data were described by frequency according to the characteristics of the evaluation index, and the *χ*^2^ test was carried out; the age was described by the mean ± standard deviation; univariate analysis and multivariate logistic regression were used to analyze the risk factors affecting pain after TACE, with *p* < 0.05 as the statistically significant difference, and a rapid screening prediction model was constructed. The receiver operating characteristic (ROC) curve was used to analyze the screening value of the prediction model for pain in patients after TACE. The predictive value was determined according to the area under the curve, and the Hosmer–Lemeshow test was used to analyze the goodness of fit of the multivariate prediction to the model in this study. The calculation of sample size in this study was mainly based on the requirement of event per variable (EPV) of at least 10 in the logistic regression equation. It is estimated that if six variables are screened in the regression equation, the number of patients required to have an event is at least 60. According to our previous experience, the probability of pain after TACE is about 35%. Considering the probability of lost follow-up is 10%, the minimum sample size required in this study is 60 / (35% × 90%) = 191.

## Results

### General condition of the patient

In total, 251 subjects were considered for potential eligibility and then 23 patients were excluded due to obvious abdominal pain symptoms before surgery (*n* = 9), a history of long-term use of analgesics (*n* = 8), and prophylactic use of analgesics (*n* = 6). A total of 228 patients with hepatocellular carcinoma who underwent TACE treatment were collected in this study, ranging in age from 34 to 87 (60.64 ± 10.24) years old. Among them, 174 were male and 54 were female (Table 1). Of all patients, 80 (35.09%) underwent the first TACE and the rest underwent two or more TACE. The majority of the population had a history of chronic liver disease (91.2%), an ECOG score of 0–1 (94.7%), and stage A in the Child–Pugh score (71.1%). 77.2% of the patients had tumors located less than 1 cm from the liver capsule, 50% of the patients had tumors larger than 5 cm, and 60.5% of the patients had one tumor. There were 68 patients with stage 1 (29.8%), 88 patients with stage 2 (38.6%), and 72 patients with stage 3 liver cancer (31.5%). The main cause of cirrhosis in patients is hepatitis B virus infection (88.4%).

**Table 1:** Baseline characteristics of study population

Variables	Study population (*n* = 228)
Age, years	60.64 ± 10.24
Male	174 (76.3)
ECOG score	
0 ~ 1	216 (94.7)
2	12 (5.3)
History of chronic liver disease	
With	208 (91.2)
Without	20 (8.8)
Child–Pugh score	
Stage A	162 (71.1)
Stage B	66 (28.9)
Tumor location	
Distance from liver capsule ≤ 1 cm	176 (77.2)
Distance from liver capsule > 1 cm	52 (22.8)
Tumor size	
< 5 cm	114 (50.0)
≧5 cm	114 (50.0)
Number of tumors	
1	138 (60.5)
≧2	90 (39.5)
CNLC	
Ia	1 (0.4)
Ib	67 (29.4)
IIa	32 (14.0)
IIb	56 (24.6)
IIIa	58 (25.4)
IIIb	14 (6.1)
Reasons for cirrhosis	
Alcohol liver	10 (4.4)
HBV	198 (86.8)
Others	16 (7.0)
Without cirrhosis	4 (1.8)

Values are expressed as mean ± standard deviation or counts (percentages)

*ECOG* Eastern Cooperative Oncology Group, *CNLC* China Liver Cancer Staging, *HBV* hepatitis B virus

### Construction of a rapid screening and prediction model for pain after TACE

The mean VAS score was 3.73 ± 2.42; meanwhile, there were 100 (43.9%) and 128 (56.1%) patients with and without post-TACE pain, respectively. As shown in Table 2, the results showed that there was no significant difference between the two groups in gender, ECOG score, history of chronic liver disease, whether there was any response after TACE, whether the target vessel was super selected during TACE, Child–Pugh score, tumor number, amount of hyper liquefied lipiodol, and whether to add embolization materials (*p* > 0.05). There were statistically significant differences between the two groups in age, tumor location, tumor size, liver cancer stage, whether to use drug-loaded microspheres, whether to use THP mixed with lipiodol for embolization, and portal vein tumor thrombosis (*p* < 0.05).

**Table 2:** Comparison of characteristics in patients with and without pain after TACE

Variables	Post-TACE pain		*p *value
	Yes (*n* = 100)	No (*n* = 128)	
Male	80 (80.0)	94 (73.4)	0.247
Age, years	58.5 ± 10.3	62.3 ± 9.9	0.004
Age ≥ 60 years	46 (46.0)	82 (64.1)	0.006
ECOG score			0.102
0 ~ 1	92 (92.0)	124 (96.9)	
2	8 (8.0)	4 (3.1)	
History of chronic liver disease	92 (92.0)	116 (90.6)	0.716
Previous post-TACE response	66 (66.0)	82 (64.1)	0.761
Intraoperative super-selected target vessels during TACE	96 (96.0)	120 (93.8)	0.450
Child–Pugh score			0.757
Stage A	70 (70.0)	92 (71.9)	
Stage B	30 (30.0)	36 (28.1)	
Tumor location			0.005
Distance from liver capsule ≤ 1 cm	86 (86.0)	90 (70.3)	
Distance from liver capsule > 1 cm	14 (14.0)	38 (29.7)	
Tumor size			0.001
< 5 cm	38 (38.0)	76 (59.4)	
≥ 5 cm	62 (62.0)	52 (40.6)	
Number of tumors			0.343
1	64 (64.0)	74 (57.8)	
≥ 2	36 (36.0)	54 (42.2)	
CNLC			< 0.001
Ia	0 (0.0)	1 (0.8)	
Ib	20 (20.0)	47 (36.7)	
IIa	8 (8.0)	24 (18.8)	
IIb	28 (28.0)	28 (21.9)	
IIIa	38 (38.0)	20 (15.6)	
IIIb	6 (6.0)	8 (6.3)	
Using drug-loaded microspheres	20 (20.0)	12 (9.4)	0.022
Dosage of ultra-liquefied lipiodol			0.301
< 10 ml	92 (92.0)	122 (95.3)	
≥ 10 ml	8 (8.0)	6 (4.7)	
THP mixed lipiodol chemoembolization	16 (16.0)	12 (9.4)	0.130
Add embolic material	80 (80.0)	94 (73.4)	0.247
PVTT	40 (40.0)	26 (20.3)	0.001

Data are expressed as *n* (%) or mean ± standard deviation

*TACE* transcatheter arterial chemoembolization, *ECOG* Eastern Cooperative Oncology Group, *CNLC* China Liver Cancer Staging, *PVTT* portal vein tumor thrombosis

After entering significantly different variables in the univariate analysis, age, tumor size, liver cancer stage, use of drug-loaded microspheres, and THP mixed with lipiodol chemoembolization were independent predictors of pain in patients after TACE (Table 3). Five independent variables were assigned: age (≥ 60 years = 1, < 60 years = 0), tumor location (> 1 cm from liver capsule = 1, distance from liver capsule ≤ 1 cm = 0), tumor size (≧5 cm = 1, < 5 cm = 0), liver cancer stage (Ia = 0, Ib = 1, IIa = 2, IIb = 3, IIIa = 4, IIIb = 5), using drug-loaded microspheres (yes = 1, no = 0), and THP combined with lipiodol chemoembolization (yes = 1, no = 0). Preliminary establishment of a predictive model for rapid screening of pain in patients after TACE: Logit (PI on pain) =  − 1.042 + 0.473 × age + 0.460 × tumor location + 2.042 × assignment of tumor size + 1.314 × liver cancer stage + 2.878 × drug-loaded microspheres + 2.605 × THP mixed iodine chemoembolization.

**Table 3:** Multivariable logistic regression analysis of factors influencing pain after TACE

		95% CI		
Variables	OR	Lower	Upper	*p *value
Age ≥ 60 years	0.473	0.263	0.849	0.012
Tumor location	0.460	0.213	0.996	0.049
Tumor size	2.042	1.114	3.741	0.021
Using drug-loaded microspheres	2.878	1.231	6.732	0.015
THP mixed lipiodol	2.605	1.068	6.356	0.035
Tumor stage	1.314	1.042	1.658	0.021

Age ≥ 60 years, tumor location, tumor size, drug-loaded microspheres, THP mixed lipiodol, PVTT, and tumor stage were included into the multivariable logistic model using the Forward: LR method

*THP* Pirarubicin hydrochloride, *OR* Odds ratio, *PVTT* Portal vein tumor thrombosis

### Effect analysis of rapid screening prediction model for pain after TACE

**Fig. 1:**
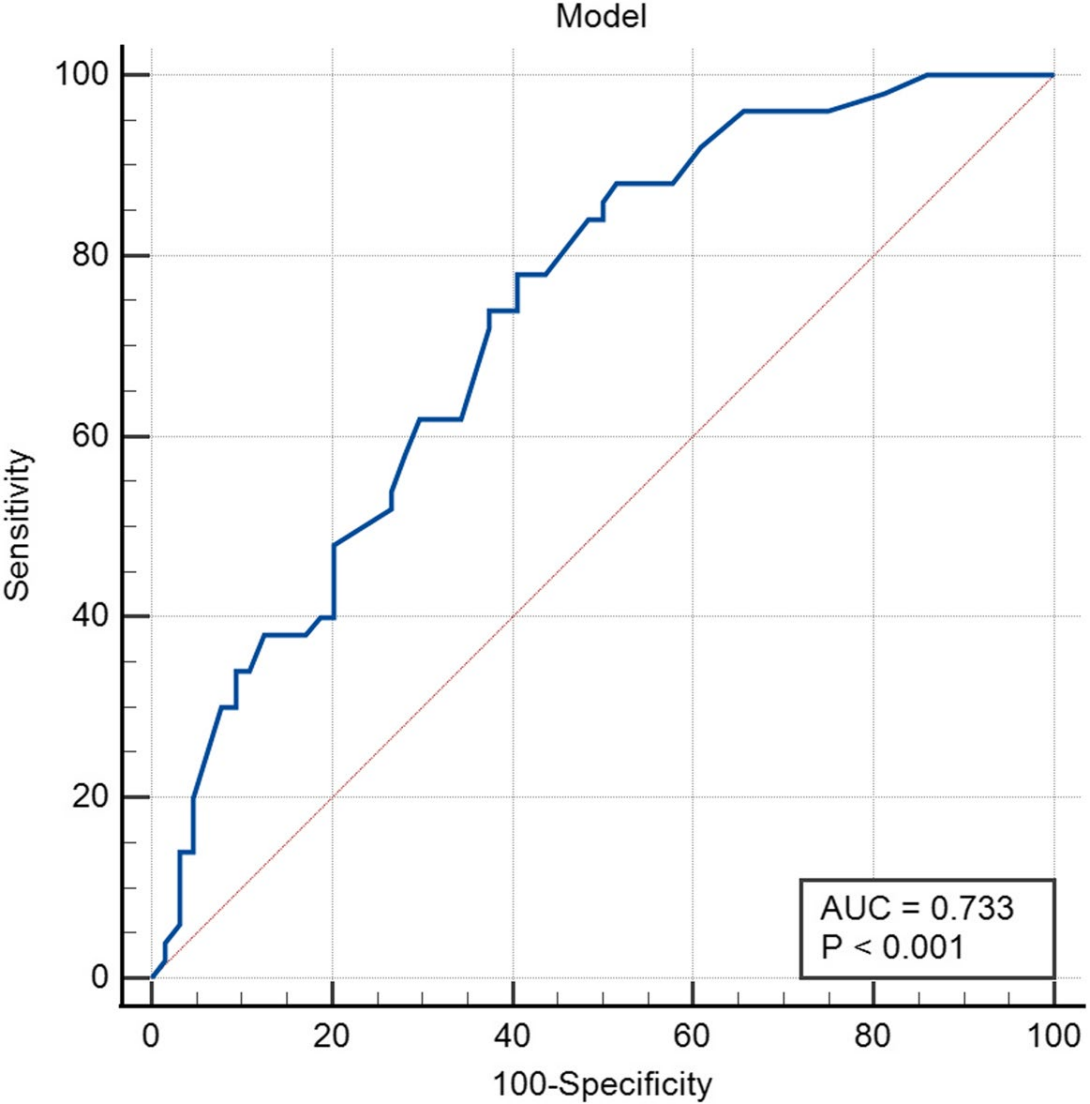
ROC curve of the pain rapid screening prediction model after TACE

The ROC curve was used to test the fitting effect of the model and the pain occurrence of patients after TACE (AUC: 0.733, 95% CI: 0.669–0.797, *p* < 0.001; see Fig. 1). The maximum value of Youden index (0.374) was used as the best critical point. The sensitivity of the model was 78.0% and the specificity was 59.4%. The results of the Hosmer–Lemeshow goodness-of-fit test showed that *χ*^2^ = 13.878, *p* = 0.085, indicating that the model has good calibration ability, indicating that the model can effectively predict the degree of pain after TACE, and it can be used for clinicians to assess the degree of pain after TACE and provide guidance for pain prediction and early intervention in patients after TACE.

## Discussion

### Construction of the questionnaire

In this study, through a comprehensive analysis of the latest domestic and foreign literature on TACE pain influencing factors, the high-risk factors for pain in TACE patients were screened out, and the items were selected based on expert opinions and feedback from clinical nurses’ application of the questionnaire. The response rates of the two rounds of expert opinions in this study were 94.1 and 100.0%, respectively, and 58.1 and 37.2% of the experts proposed revisions. Experts who participated in this research consultation were all associate chief physicians or above who are engaged in TACE treatment of primary liver cancer. Among them, 81.75% (13/16) had master’s degree or above and 93.7% (15/16) of experts have more than 10 years of work experience.

Postoperative pain will increase the unpleasant experience of patients with TACE treatment, give patients a certain amount of negative reinforcement, and affect the next treatment. In this study, there were 100 patients (100/228) with pain after TACE, and the incidence rate was similar to the results of domestic and foreign studies [2–4]. So far, potentially beneficial interventions for pain management in TACE patients include intra-arterial infusion of lidocaine before embolization, preoperative oral or intravenous analgesics, wrist–ankle acupuncture, etc. However, these methods all have certain shortcomings. Therefore, in order to effectively address the pain of patients, a systematic approach to comprehensive treatment is necessary [10].

### Analysis of the influencing factors of pain in patients after TACE

In this study, 228 TACE patients admitted to our hospital were analyzed and found that tumor size, liver cancer stage, use of drug-loaded microspheres, and THP mixed with lipiodol chemoembolization were independent predictors of pain in patients after TACE. In this study, the incidence of pain after embolization with drug-loaded microspheres is consistent with the related research results of some scholars at home and abroad [11–13]. Some scholars have found that the level of abdominal pain in TACE patients undergoing embolization using adriamycin-eluting microspheres is significantly lower than that in patients undergoing TACE using conventional methods [14, 15]. However, the effect of using CalliSpheres® drug-loaded microspheres to embolize tumor feeding arteries on pain after TACE is controversial. Zhang et al. [16] conducted a multi-center retrospective study and found that the incidence of pain in patients with traditional TACE was higher than that with drug-loaded microspheres. Larger liver tumor is an influencing factor for pain after TACE. The results of this study showed that tumor size was positively correlated with the occurrence of pain, so tumor size was also an independent predictor of pain. This is different from the research results of foreign scholars Benzakoun et al. [17]. In addition, theoretically, the closer the tumor is to the liver capsule, the more pronounced the pain is after embolization. However, related clinical studies have shown different results.

**Fig. 2:**
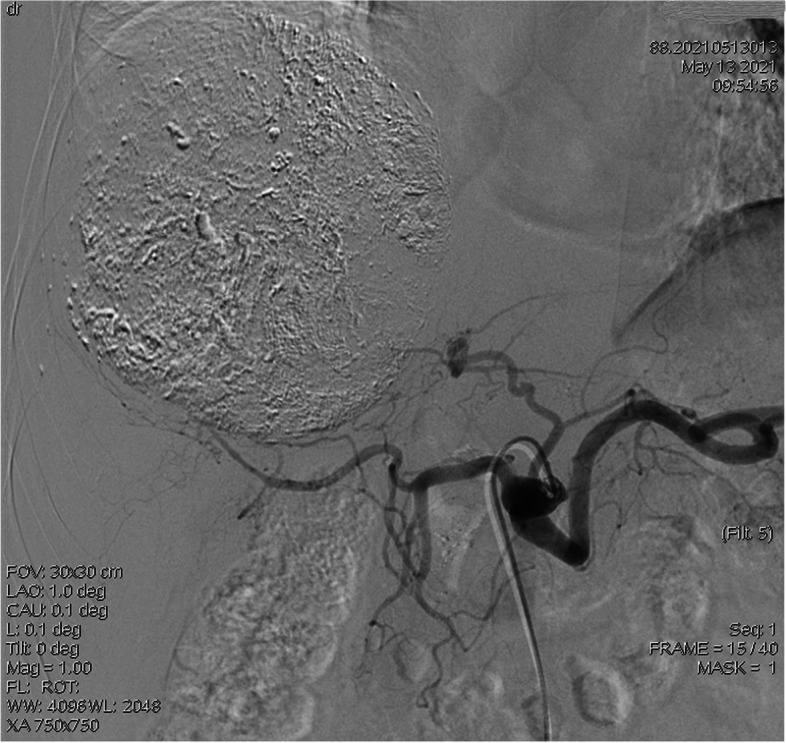
Angiographic images of patients with sufficient embolization

**Fig. 3:**
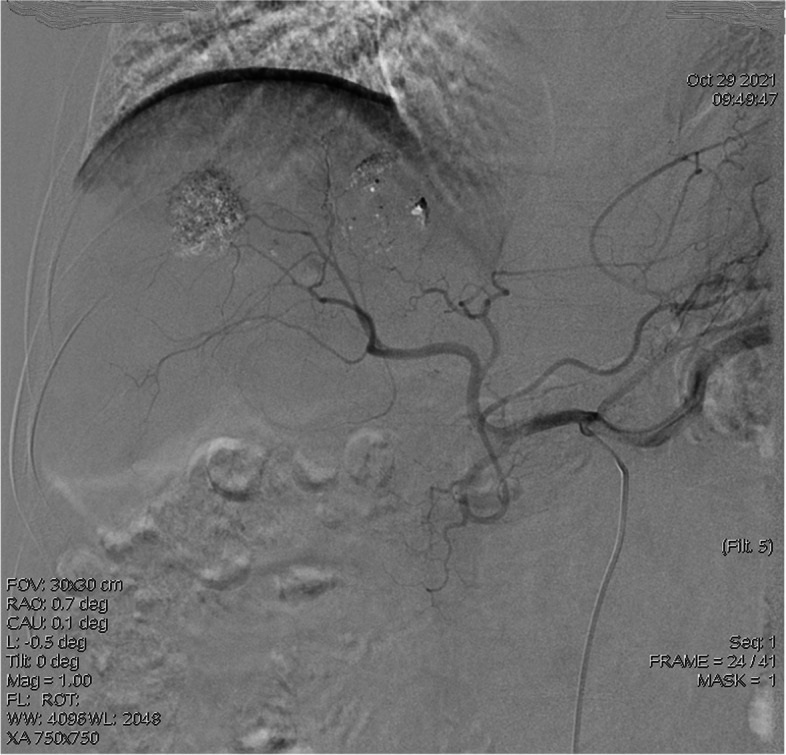
Angiographic images of patients with insufficient embolization

Liver cancer stage (CNLC) is a risk factor for pain after TACE. The staging of liver cancer is very important for prognostic evaluation. There are many staging schemes abroad. In this study, the Chinese staging scheme for liver cancer (CNLC) was included as an influencing factor. The results of this study showed that patients with stage IIIa had the highest incidence of pain after TACE, which may be related to vascular invasion, chemotherapy, or tumor embolization in stage IIIa patients. In addition, when the tumor invades blood vessels, the interventional doctor is stricter in the degree of embolization. In clinical observation, it is found that the patients with adequate embolization have more significant pain than those with insufficient embolization (see Figs. 2 and 3). Embolization of THP mixed with lipiodol is an influencing factor of postoperative pain after TACE. Chemoembolization with THP mixed with lipiodol is a combination of two ways to eliminate tumors. THP has the effect of reducing tumor activity in patients with primary liver cancer [18], but THP can also make patients feel severe pain. Through observation and statistical analysis, this study found that THP mixed with lipiodol is more prone to pain than other drugs mixed with lipiodol for chemoembolization, which is consistent with the results of Luo et al. [3].

### Analysis of the effect and limitations of the rapid screening prediction model for pain after TACE

In this study, through the univariate analysis of pain in patients after TACE, a rapid screening prediction model for pain in patients after TACE was initially established. Then, the ROC curve was used to test the fitting effect of the model and the pain occurrence of patients after TACE. The area under the ROC curve of the rapid screening prediction model for pain after TACE constructed in this study was calculated to be 0.733, which has good sensitivity and specificity. Moreover, it is found that the model has better calibration ability through inspection. At the same time, the independent influencing factors obtained in this study are partially similar to the conclusions of other scholars in the past [19, 20].

However, due to the limited time and the scope of the investigation, the consultant experts invited by this study are not from a wide range of sources, and the survey samples are relatively insufficient. Rapid screening and prediction of pain after TACE has not been performed clinically, but only based on the previously published literature data. Analysis and verification showed that the predicted results of the model may still have some discrepancies with the current incidence of clinical pain after TACE. In this study, we used logistic regression equation to make the model, mainly to ensure the accuracy of the data, because scoring systems and other types are only rough estimates of risk. Larger subsequent studies are warranted to validate the preliminary results in the present study and develop user-friendly models such as web-based calculators. In the later stage, the corresponding software will be developed based on the model. At the same time, with the help of information technology and port docking, the corresponding information can be easily extracted, the risk value can be quickly calculated, and more case information can be collected at the same time to verify the prediction conclusion of the model, providing guidance for pain prediction and intervention. We did not evaluate the potential impact of anti-anxiety drugs (flupentixol melitracine) on the performance of the model for the prediction of TACE pain. There remains uncertainty regarding whether the length of pain and the degree of analgesics use contribute to post-TACE pain, which needs further studies to address these questions.

In summary, this study analyzed 228 TACE patients admitted to our hospital and related literature published in the past 5 years. The analysis results showed that tumor size, liver cancer stage, drug-loaded microspheres, and THP combined with lipiodol chemoembolization were independent predictors of pain in patients after TACE. Although some predictors were reported in previous studies, we validated the association of these predictors with post-TACE pain in our Chinese population. In addition, we constructed a novel prediction model by combining these independent predictors, which is a simple and useful tool for clinical decision-making. The rapid screening prediction model for pain in patients after TACE based on these four factors has been verified to have certain predictive power. In addition, these four risk factors can be quickly extracted and evaluated through information sharing on the hospital intranet before surgery. Clinicians can use the rapid screening prediction model of this study to comprehensively evaluate the influencing factors of pain in patients at an early stage and can effectively predict the degree of pain of patients after surgery, relieve postoperative pain of patients, and provide effective guidance for clinical pain prediction and intervention after TACE.

## Data Availability

All data generated or analyzed during this study are included in this article.
